# Development of epithelial cholinergic chemosensory cells of the urethra and trachea of mice

**DOI:** 10.1007/s00441-021-03424-9

**Published:** 2021-02-22

**Authors:** Alexander Perniss, Patricia Schmidt, Aichurek Soultanova, Tamara Papadakis, Katja Dahlke, Anja Voigt, Burkhard Schütz, Wolfgang Kummer, Klaus Deckmann

**Affiliations:** 1 Institute for Anatomy and Cell Biology, German Center for Lung Research (DZL), Excellence Cluster Cardiopulmonary Institute (CPI), Justus-Liebig-University Giessen, Giessen, Germany; 2 Department of Gastroenterology, Infectious Diseases and Rheumatology, Charité Universitätsmedizin Berlin, Campus Benjamin Franklin, Berlin, Germany; 3 Max Rubner Laboratory, German Institute of Human Nutrition (DIfE), Nuthetal, Germany; 4 Institute of Anatomy and Cell Biology, Philipps-University, Marburg, Germany

**Keywords:** Brush cells, Tuft cells, Innate immunity, Toll-like receptors, MyD88, Solitary chemosensory cells, Chemosensation

## Abstract

Cholinergic chemosensory cells (CCC) are infrequent epithelial cells with immunosensor function, positioned in mucosal epithelia preferentially near body entry sites in mammals including man. Given their adaptive capacity in response to infection and their role in combatting pathogens, we here addressed the time points of their initial emergence as well as their postnatal development from first exposure to environmental microbiota (i.e., birth) to adulthood in urethra and trachea, utilizing choline acetyltransferase (ChAT)-eGFP reporter mice, mice with genetic deletion of MyD88, toll-like receptor-2 (TLR2), TLR4, TLR2/TLR4, and germ-free mice. Appearance of CCC differs between the investigated organs. CCC of the trachea emerge during embryonic development at E18 and expand further after birth. Urethral CCC show gender diversity and appear first at P6-P10 in male and at P11-P20 in female mice. Urethrae and tracheae of MyD88- and TLR-deficient mice showed significantly fewer CCC in all four investigated deficient strains, with the effect being most prominent in the urethra. In germ-free mice, however, CCC numbers were not reduced, indicating that TLR2/4-MyD88 signaling, but not vita-PAMPs, governs CCC development. Collectively, our data show a marked postnatal expansion of CCC populations with distinct organ-specific features, including the relative impact of TLR2/4-MyD88 signaling. Strong dependency on this pathway (urethra) correlates with absence of CCC at birth and gender-specific initial development and expansion dynamics, whereas moderate dependency (trachea) coincides with presence of first CCC at E18 and sex-independent further development.

## Introduction

Referring to their apical brush or tuft of stiff microvilli, identified in ultrastructural studies, a group of rare solitary epithelial cells has been initially termed brush or tuft cells (Rhodin and Dalhamn 1956; Sbarbati and Osculati 2005). Meanwhile, structural, histochemical, and single cell sequencing data revealed several unique characteristics defining distinct cell populations within and among organs (Deckmann et al. 2014; Krasteva et al. 2011; Montoro et al. 2018; Nadjsombati et al. 2018; Yamamoto et al. 2018). One of them is characterized by the expression of the acetylcholine synthesizing enzyme, choline acetyltransferase (ChAT), and the expression of the taste transduction signaling cascade, including the taste-specific G protein α-gustducin (GNAT3), phospholipase Cβ2 (PLCβ2), and the transient potential receptor cation channel subfamily M (melanostatin) member 5 (TRPM5). Such cholinergic chemosensory cells (CCC) have been identified in the upper airways (Saunders et al. 2014; Tizzano et al. 2011), the vomeronasal organ (Ogura et al. 2010), the auditory tube (Krasteva et al. 2012b), the trachea (Krasteva et al. 2011), the conjunctiva (Wiederhold et al. 2015), the gastro-intestinal tract including the gall bladder (Schutz et al. 2015), the urethra (Deckmann et al. 2014), and the thymus (Panneck et al. 2014). All cells of this category are closely related, even though there are organ-specific characteristics (Deckmann and Kummer 2016; Finger and Kinnamon 2011; Nadjsombati et al. 2018; O’Leary et al. 2019).

Their function is only partially identified. They utilize elements of the canonical taste transduction cascade to respond to potentially harmful substances including bacterial products (Deckmann et al. 2014; Finger et al. 2003; Krasteva et al. 2012a; Ogura et al. 2011; Saunders et al. 2014; Sbarbati et al. 2009; Tizzano et al. 2010) and helminths (Gerbe et al. 2016; Howitt et al. 2016; Nadjsombati et al. 2018; von Moltke et al. 2016). Upon activation, CCC initiate protective mechanisms like reflex inhibition of inspiratory activity (Krasteva et al. 2011; Tizzano et al. 2010) or stimulation of micturition (Deckmann et al. 2014), initiation of neurogenic inflammation (Saunders et al. 2014), and triggering type 2 immune responses (Bankova et al. 2018; Gerbe et al. 2016; Howitt et al. 2016; Nadjsombati et al. 2018; von Moltke et al. 2016). Accordingly, they have been attributed with a sentinel function and are regarded as crucial elements of the mucosal innate immune system (Krasteva and Kummer 2012; Kummer and Deckmann 2017; Middelhoff et al. 2017; Schneider et al. 2019; Tizzano and Finger 2013).

The transcription factor Skn-1a/Pou2f3 is required for CCC development, and its genetic deletion results in their absence in several organs (Ohmoto et al. 2013; Yamashita et al. 2017). Unlike taste cells in the taste buds, neither development nor maintenance of nasal CCC is dependent on intact innervation (Gulbransen et al. 2008). In postnatal life, CCC do undergo turnover like the surrounding epithelium, as initially demonstrated for the nose (Gulbransen and Finger 2005), and respond dynamically to challenges. While tracheal CCC have been described as “a stable population in a dynamic epithelium” under unchallenged conditions (Saunders et al. 2013), they increase markedly in numbers after exposure to house dust mites or mold in a leukotriene-dependent manner (Bankova et al. 2018). Severe infection with H1N1 influenza virus even provokes de novo appearance of solitary chemosensory cells (cholinergic traits not investigated in this study) in the murine distal lung, whereas such cells are not present in uninfected lungs (Rane et al. 2019). In the intestine, their number increases vastly in response to helminth and protozoan infection (Gerbe et al. 2016; Howitt et al. 2016; Nadjsombati et al. 2018; Schneider et al. 2018; von Moltke et al. 2016). At least in the intestine, CCC not only increase in numbers during infection but are also necessary for helminth clearance. Mice lacking TRPM5 remain infected with *Nippostrongylus brasiliensis*, whereas wildtype mice expel this helminth within 2 weeks (Nadjsombati et al. 2018). Given their adaptive capacity in response to infection and their role in combatting pathogens, we addressed the time points of their initial emergence as well as their postnatal development from first exposure to environmental microbiota (i.e., birth) to adulthood, utilizing ChAT-eGFP reporter mice and focusing upon tracheal, urethral, and, to a lesser extent, thymic CCC. The necessity of direct contact to living microbiota for the development of CCC was assessed by using germ-free mice. Since these mice are still exposed, e.g., via the food, to bacterial products and bacterial remains capable of triggering innate immune responses by activating toll-like receptors (TLRs), we also included MyD88 (myeloid differentiation primary response 88) knockout (KO) mice in this study. MyD88 is involved in downstream signaling of all TLRs, except TLR3, as well as the interleukin-1-receptor (Wang et al. 2014). As genetic loss of MyD88 indeed resulted in lower CCC numbers, we further included TLR2 and TLR4 single- and double-deficient mice to narrow down the spectrum of signaling pathways potentially being involved. TLR2 and TLR4 were chosen because of their well-known importance in the recognition of bacterial lipopeptides and lipoteichoic acids of Gram-positive bacteria in the case of TLR2 (Irvine et al. 2013) and lipopolysaccharides of Gram-negative bacteria in the case of TLR4 (Park et al. 2009).

## Material and methods

### Animals

ChAT-eGFP (B6.Cg-Tg(RP23-268L19-EGFP)2Mik/J; Stock No. 007902) mice were obtained from Jackson Laboratory (Bar Habor, ME, USA). A second ChAT-eGFP mouse strain (von Engelhardt et al. 2007) was exclusively used to assess CCC appearance during embryonic development. Mice were housed in the animal facilities of the Justus-Liebig-University Giessen or the Philipps-University Marburg under specific-pathogen-free (SPF) conditions (10 h dark, 14 h light), with free access to food and water.

Germ-free mice (C57BL/6N; germ-free; > 12 weeks) were obtained from the gnotobiotic animal facility of the German Institute of Human Nutrition, Potsdam-Rehbruecke, Germany. Gnotobiotic mice were maintained in positive‐pressure isolators. Mice were housed individually in polycarbonate cages on irradiated wood chips (25 kGy) at 22 ± 2 °C and a relative humidity of 55 ± 5% on a 12 h light-dark cycle. All mice had unrestricted access to irradiated (50 kGy) experimental diets and autoclaved water throughout the experiment. Control mice (C57BL/6N, same sex and age as germ-free mice; SPF) were obtained from Jackson Laboratory and housed at the animal facility of the Justus-Liebig-University Giessen in individually ventilated cages under SPF conditions. TLR2-KO (C57BL/10ScSn-TLR2^tm1^), TLR4-def (C57BL/10ScN-TLR4 (spontaneous deletion of the *Trl4* gene)), and TLR2-KO/TLR4-def (C57BL10ScN-TLR4/TLR2^tm1^) and corresponding wildtypes (TLR-WT; C57BL/10ScSn) were also obtained from the animal facility of the German Institute of Human Nutrition, Potsdam-Rehbruecke, Germany; all investigated mice of this strains were > 12 weeks old. These mice were bred as described previously (Heimesaat et al. 2007).

Tissues from MyD88-KO mice (B6.129-Myd88^tm1^Aki; > 12 weeks) and corresponding wildtypes (MyD88-WT; litter mates of MyD88-KO mice) were obtained from two independent sources. The strain originally generated by Adachi et al. (1998) was bred and provided by Rainer Glauben, Charite Berlin, and by Axel Pagenstecher, Philipps-University Marburg.

This study was carried out in accordance with the recommendations of European Communities Council Directive of 24th November 1986 (86/609/EEC). The protocol was approved by the local authorities, i.e., Regierungspräsidium Giessen, Germany (reference no. 572_M, 571_M, 557_AZ, Ex-15–2018), the Office for Agriculture, Ecology, and Regional Planning of the State of Brandenburg (Germany) according to §8.1 Animal Welfare Act (approval number: 23-2347-6-2009 and 23-2347-24-2010), and the Landesamt für Gesundheit und Soziales (LAGESO), Berlin (approval number T0294/10).

### 3R statement

In this study, more than 450 tissue specimens were collected, investigated, and analyzed. In order to adhere to the 3R principle (reduction, replacement, and refinement in animal experiments principle (Russell and Burch 1959)) the number of animals used for this study was kept to a minimum by taking multiple organs (urethra, trachea, thymus) from the same animal and by taking specimens from animals that have been sacrificed for other purposes.

### Immunohistochemistry and whole-mount immunostaining

Organs used for immunohistochemistry and whole-mount immunostaining were either freshly dissected and fixed by immersion or taken from animals fixed by transcardiac perfusion with 4% paraformaldehyde (in 0.1 M phosphate buffer, pH 7.4; both purchased from Carl Roth, Karlsruhe, Germany) or Zamboni solution (2% paraformaldehyde/15% saturated picric acid; Merck, Darmstadt, Germany, in 0.1 M phosphate buffer, pH 7.4), preceded by a flush with vascular rinsing solution (Forssmann et al. 1977). Organs were washed in 0.1 M phosphate buffer and embedded in paraffin (Paraplast Plus®, Leica, Nussloch, Germany) or incubated overnight in 18% sucrose (Carl Roth, Karlsruhe, Germany) in 0.1 M phosphate buffer, embedded in Tissue-Tek® O.C.T.™ Compound (Sakura Finetek Germany GmbH, Staufen, Germany) and frozen in liquid nitrogen. For immunostainings, tissue sections or whole-mounts of investigated strains were processed simultaneously with corresponding controls, e.g., wildtypes. Primary antibody was applied to 4–18 tissue sections (frozen 10 µm or paraffin 5 µm) from every organ. At least three animals per experimental setup were analyzed. For whole-mount immunostainings of tracheae, the trachea was dissected from the larynx to the bifurcation and the trachealis muscle was cut longitudinally. For whole-mount immunostainings of urethrae, the whole urethra was taken and cut longitudinally. The tracheae and urethrae were opened and fixed flat on a silicon elastomer (Dow Corning, Midland, MI, USA) with insect needles (Fiebig-Lehrmittel, Berlin, Germany). Specimens were permeabilized with 0.3% Triton X 100 (Carl Roth, Karlsruhe, Germany) for 2 h; unspecific protein binding sites were saturated by incubation with 4% horse serum (PAA Laboratories Inc., Pasching, Austria) and 1% bovine serum albumin (Sigma Aldrich/Merck, Darmstadt, Germany) in 0.005 M phosphate buffer for 2 h. Samples were incubated in primary and secondary antibodies overnight each, rinsed, post-fixed for 10 min in 4% paraformaldehyde, and mounted in Mowiol (Sigma Aldrich/Merck, Darmstadt, Germany) containing 4′,6-diamidino-2-phenylindol (DAPI, 1 µg/ml, Sigma Aldrich/Merck, Darmstadt, Germany).

To investigate prenatal development of CCC, embryos of ChAT-eGFP mice (von Engelhardt et al. 2007) were harvested at specified embryonic stages (E12, *N* = 4; E14, *N* = 5; E16, *N* = 8; E18, *N* = 6) and shortly after birth (P0, *N* = 3), which were determined by fertilization. After extraction, embryos were sacrificed, fixed by immersion with Zamboni solution, cut into halves, and embedded in paraffin. Embryos were sectioned (5 µm), and every 10th section was stained with H.E. or Giemsa for orientation. Every second section harboring tracheal epithelium was immunostained and analyzed.

Primary antibodies were chicken-anti-GFP (green fluorescent protein, NB100-1614, 1:4000 dilution; Novus Biologicals, Centennial, USA), goat-anti-ChAT (AB144P, 1:250 dilution; Merck Millipore/Merck, Darmstadt, Germany), rabbit-anti-DCAMKL1 (Serine/threonine-protein kinase DCLK1 (Doublecortin-like kinase 1)), ab31704 (1:2000 dilution; Abcam, Cambridge, UK), and rabbit-anti-TPRM5 (1:4000–8000 (Kaske et al. 2007)).

Secondary antibodies were donkey-anti-chicken IgG conjugated to fluorescein isothiocyanate (FITC; 703-095-155; 1:800; Dianova, Hamburg, Germany), donkey-anti-rabbit IgG conjugated to Cyanine 3 (Cy3; 2567112; 1:2000; Merck Millipore/Merck, Darmstadt, Germany), donkey-anti-rabbit IgG Alexa 488 (A21206; 1:500; Thermo Fisher Scientific Inc., Waltham, MA, USA), donkey-anti-goat IgG Alexa 488 (A11055; 1:1000; Thermo Fisher Scientific Inc., Waltham, MA, USA), and donkey-anti-goat IgG Cy3 (AP180C; 1:16,000; Merck Millipore/Merck, Darmstadt, Germany). Specificity of secondary reagents was validated by omission of primary antibodies.

CCC number in ChAT-eGFP mice was assessed by enhancement of endogenous eGFP signal with antibodies against eGFP. In C57BL/6N-mice, MyD88-KO mice, TLR-KO mice, TLR-def mice, and corresponding wildtypes, all not carrying the ChAT-eGFP reporter, CCC numbers were assessed by TRPM5-, ChAT-, or DCAMKL1-immunolabeling. To evaluate the degree of co-localization of ChAT-eGFP expression and TRPM5-immunoreactivity in CCC, the TRPM5-antibody was applied to whole-mounts (trachea and urethra) from ChAT-eGFP mice.

Sections and whole-mounts were evaluated by epifluorescence microscopy (Axioplan 2, Zeiss, Oberkochen, Germany) or with a confocal laser scanning microscope (LSM 710, Zeiss, Oberkochen, Germany). Overlay images were created using ImageJ (https://imagej.nih.gov/ij). For evaluation of cell numbers in tracheae and urethrae, we used whole-mount preparations and counted all positive cells in the organ manually or interactively using an ImageJ cell counter plug-in. Counting of a data set was performed by the same person to exclude experimenter dependent bias.

Surface area of trachea and urethra was calculated using ImageJ, except for adult tracheae of germ-free, control mice, MyD88-KO and MyD88-WT. In these cases, 8 pictures at × 10 magnification were taken along tracheal whole-mounts as illustrated in Supplementary Fig. 1a and used to evaluate CCC number per square millimeter. The number of CCC in TLR-deficient mice and corresponding wildtypes was evaluated using frozen or paraffin sections; here, the number of positive cells per millimeterbasal lamina was calculated utilizing the software Axiovision (Zeiss, Oberkochen, Germany) in case of the trachea (at least 10 longitudinal sections per animal were analyzed) or positive cells per section for the urethra (on average 25 sections per animal) were counted. To investigate appearance of CCC in thymi of different ages, at least 6 sections per animal (*N* = 3, mixed sexes) were analyzed.

**Fig. 1:**
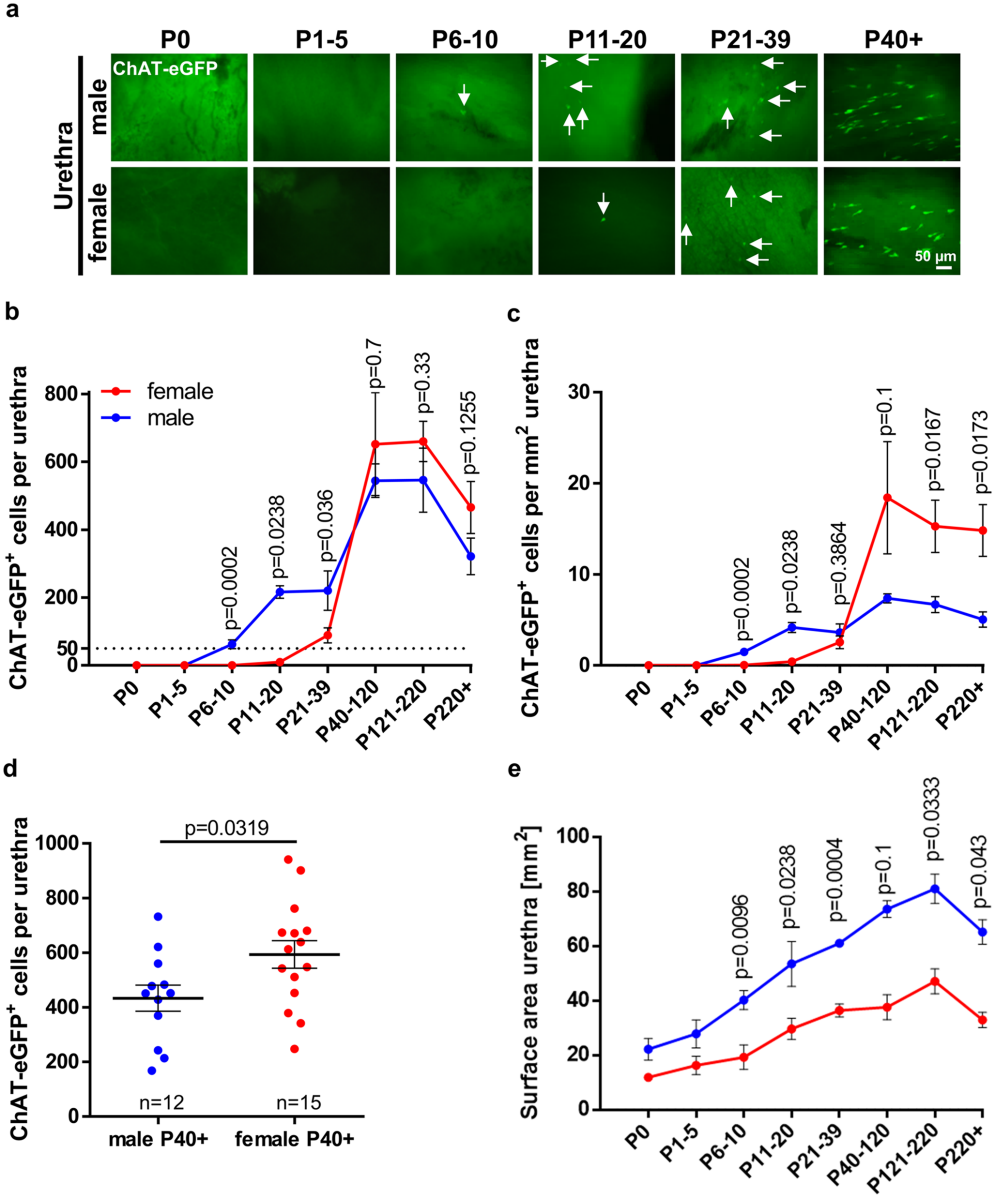
Postnatal development of CCC in the urethra. **a** Immunofluorescence using tissue from ChAT-eGFP mice, eGFP signal was antibody enhanced, representative images of female and male urethrae at different time points during postnatal development; arrows mark ChAT-eGFP-positive urethral CCC. **a** and **b** Quantitative analysis of ChAT-eGFP-positive cells per urethra **b** and per mm^2^ **c** using whole-mount preparations in female and male mice at different time points during development; male: P0 *N* = 5; P1-5 *N* = 8; P6-10 *N* = 17; P11-20 *N* = 3; P21-39 *N* = 6; P40-120 *N* = 3; P121-220 *N* = 3; P250 + * N* = 6; female: P0 *N* = 3; P1-5 *N* = 4; P6-10 *N* = 6; P11-20 *N* = 6; P21-39 *N* = 9; P40-120 *N* = 3; P121-220 *N* = 7; P220 + * N* = 5. Dashed line marks cell count of 50 cells per urethra. **d** Numbers of ChAT-eGFP-positive cells per urethra in animals P40+ , cumulative data of the three oldest age groups depicted in **b**; **e** Surface area of male and female urethrae used for generating **c**. **b**–**e** Graphs depict means and SEM. Blue: males, red: females. P-values were calculated with Mann-Whitney test

### Statistical analysis

Data were analyzed by Mann-Whitney test or Kruskal-Wallis test with GraphPad Prism 7 (GraphPad Software Inc., La Jolla, CA, USA). *P* ≤ 0.05 were regarded as statistically significant.

## Results

### Sexual dimorphism of postnatal urethral CCC development

The time course of urethral CCC appearance showed a sexual dimorphism in ChAT-eGFP mice (Fig. 1). In male mice, urethral CCC appeared first between P6 and P10. In female mice, urethral CCC appeared first between P11 and P20. In both sexes, maximum urethral CCC numbers were reached between P40 and P220 and declined thereafter (Fig. 1b and c). Male mice had significantly higher absolute CCC numbers and CCC density per surface area than females in the periods P6–10, P11–20, and P21–39. In older animals (P40+), however, urethral CCC were more abundant in female mice (Fig. 1b, c, d; Supplementary Fig. 2). This gender difference was particularly pronounced in urethral CCC density, since the total urethral surface area was about 1.8 times larger in males than in females (Fig. 1e).

**Fig. 2:**
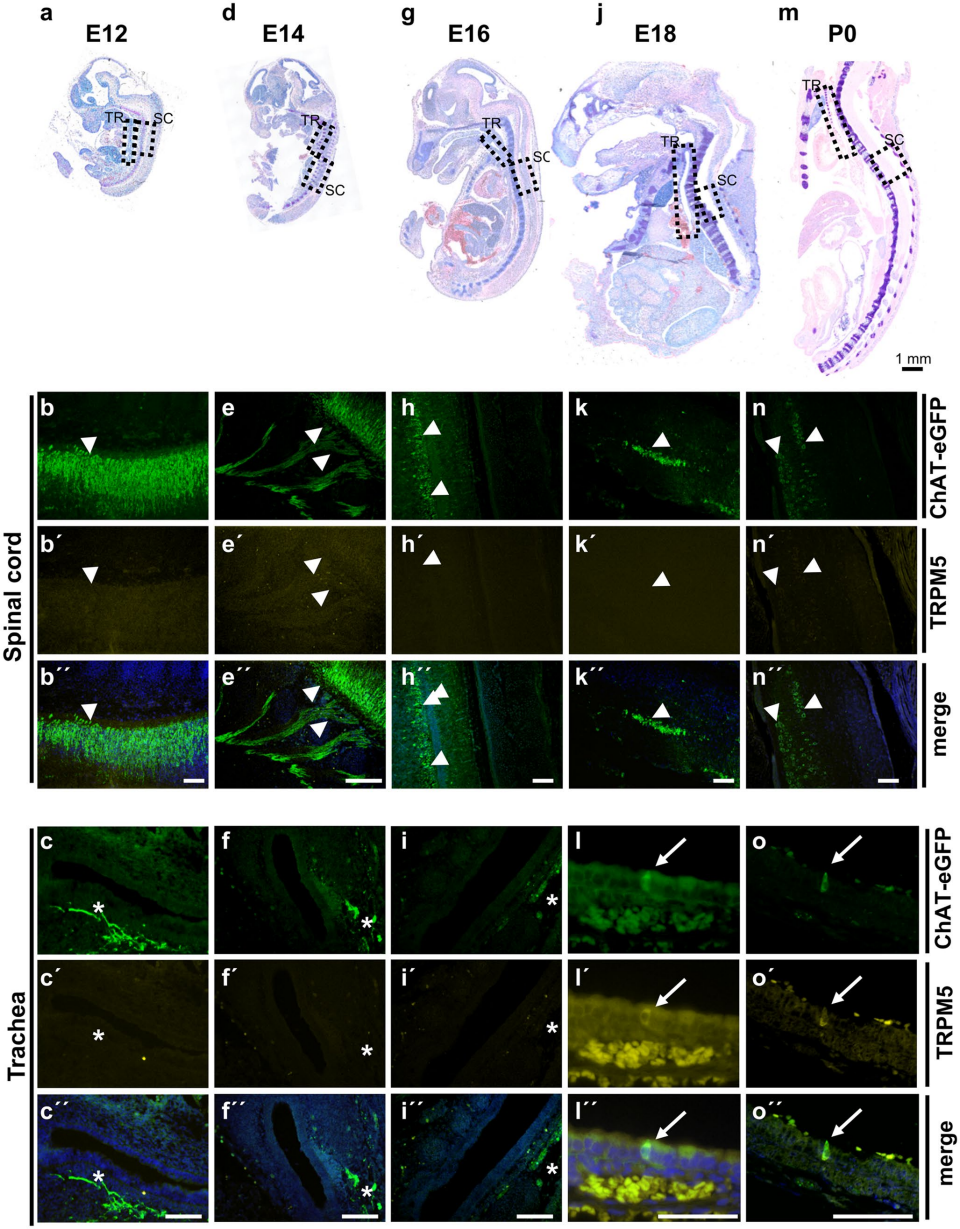
Prenatal and perinatal development of tracheal CCC. Giemsa-stained paraffin sections from ChAT-eGFP mice (von Engelhardt et al. 2007) at distinct developmental stages with corresponding immunofluorescence images of trachea and spinal cord **a**–**c** E12 (*N* = 4), **d**–**f** E14 (*N* = 5), **g**–**i** E16 (*N* = 8), (j–l) E18 (*N* = 6), and (m–o) P0 (*N* = 3). Dashed boxes highlight locations where according immunofluorescence images of trachea (TR; **b**, **e**, **h**, **k**, and **n**) and spinal cord (SC; **c**, **f**, **i**, **l**, and **o**) were taken from adjacent sections double-labeled for TRPM5 and GFP; arrows mark ChAT-eGFP- and TRPM5-positive tracheal CCC at E18 and P0; arrowheads mark ChAT-eGFP-positive neurons; asterisks mark ChAT-eGFP-positive nerve fibers below the tracheal epithelium. Scale bar depicts 100 µm in the immunofluorescence images and 1 mm in Giemsa-stained overview images

### CCC of the trachea appear already during embryonic development

**Fig. 3:**
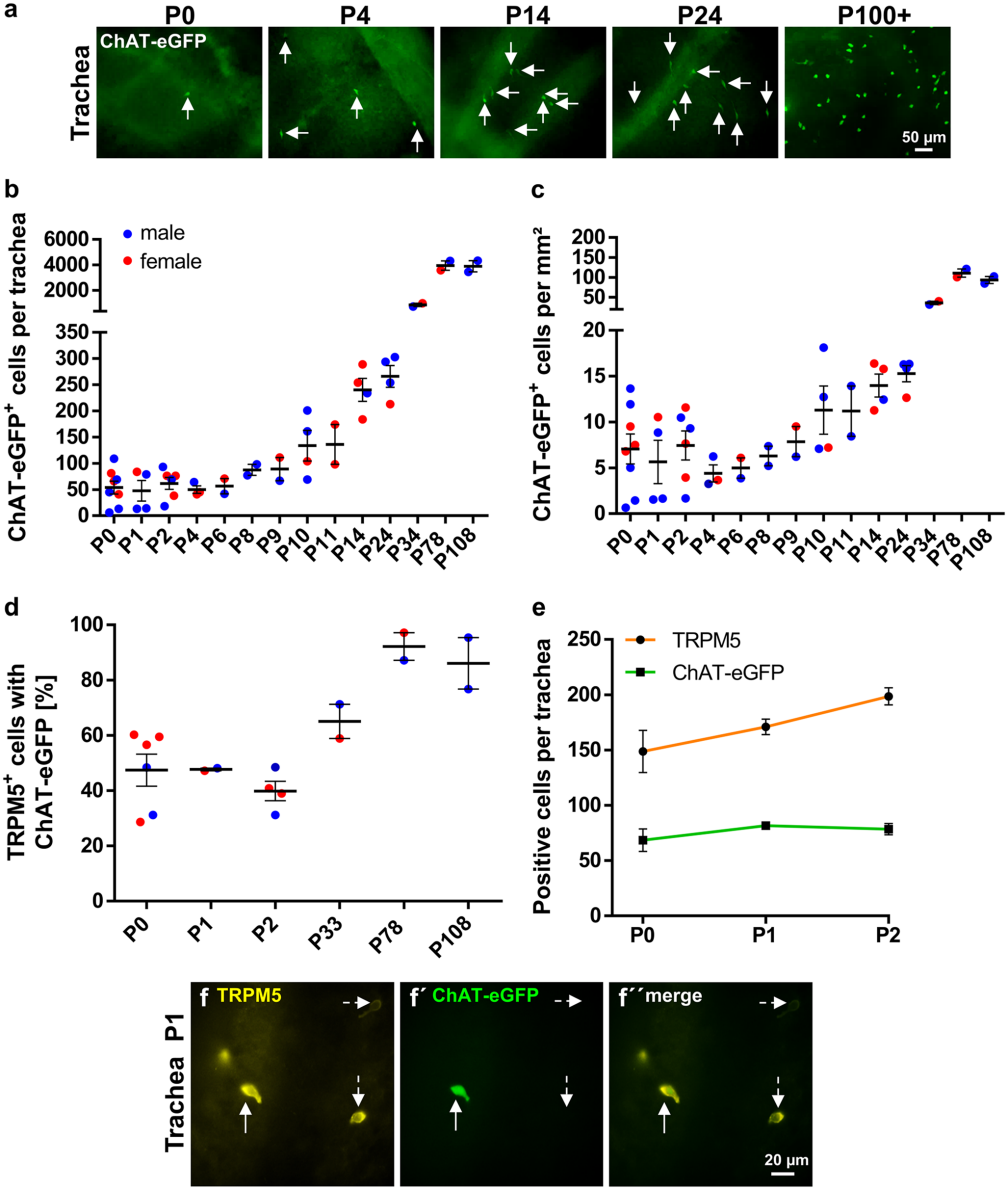
Postnatal development of CCC in the trachea. **a** Immunofluorescence with GFP antibody using tissue from ChAT-eGFP mice, representative images of tracheae at different time points during postnatal development; arrows point to ChAT-eGFP-positive tracheal CCC. GFP-positive cells were counted in tracheal whole-mount preparations; absolute numbers are given in **b**, cell number per mm^2^ is shown in **c**. **d** Percentage of TRPM5-positive cells which also were positive for ChAT-eGFP from same animals as in **b** and **c**. **e** Total number of TRPM5- and ChAT-eGFP-positive cells at P0–P2 from same animals as in **b** and **c** (*N* = 2–6 each time point). **f** Representative immunofluorescence pictures with antibodies against TRPM5 and GFP of a tracheal whole-mount from an one day old ChAT-eGFP animal; dashed arrows mark a TRPM5 single-positive cell; solid arrows mark a TRPM5 and ChAT-eGFP double-positive cell. Graphs depict mean and SEM; **b**–**d** all data points depicted in scatter plots. Blue: males, red: females

In ChAT-eGFP mice (von Engelhardt et al. 2007), CCC could not be detected within the tracheal epithelium at E12, E14, and E16. They appeared first at E18 in 5 of 6 investigated tracheae (Fig. 2), albeit in rare occurrence compared with pups sacrificed directly after birth (P0) (Figs. 2 and 3). Postnatal development was quantified in ChAT-eGFP (B6.Cg-Tg(RP23-268L19-EGFP)2Mik/J) mice. The number and density of tracheal CCC increased considerably until P78 (from 54 at P0 to 3950 cells at P78, Fig. 3a–c and Supplementary Fig. 1b and 3). In contrast to the urethra, no gender difference could be observed (Fig. 3b, c; Supplementary Fig. 1c). In tracheal whole-mount preparations of adult mice, about 89% of the cells labeled with TRPM5 antibody were also ChAT-eGFP-positive (Fig. 3d; Supplementary Fig. 3). In neonatal animals (P0-P2), however, only 45% of cells were double-positive (*p* < 0.0004, Mann-Whitney test). This co-localization increased further to 65% at P33 (Fig. 3e and f). The number of cells only positive for eGFP was extremely low in the tracheal epithelium throughout all ages.

### TLR2/TLR4 and MyD88 deficiencies impair postnatal CCC expansion

**Fig. 4:**
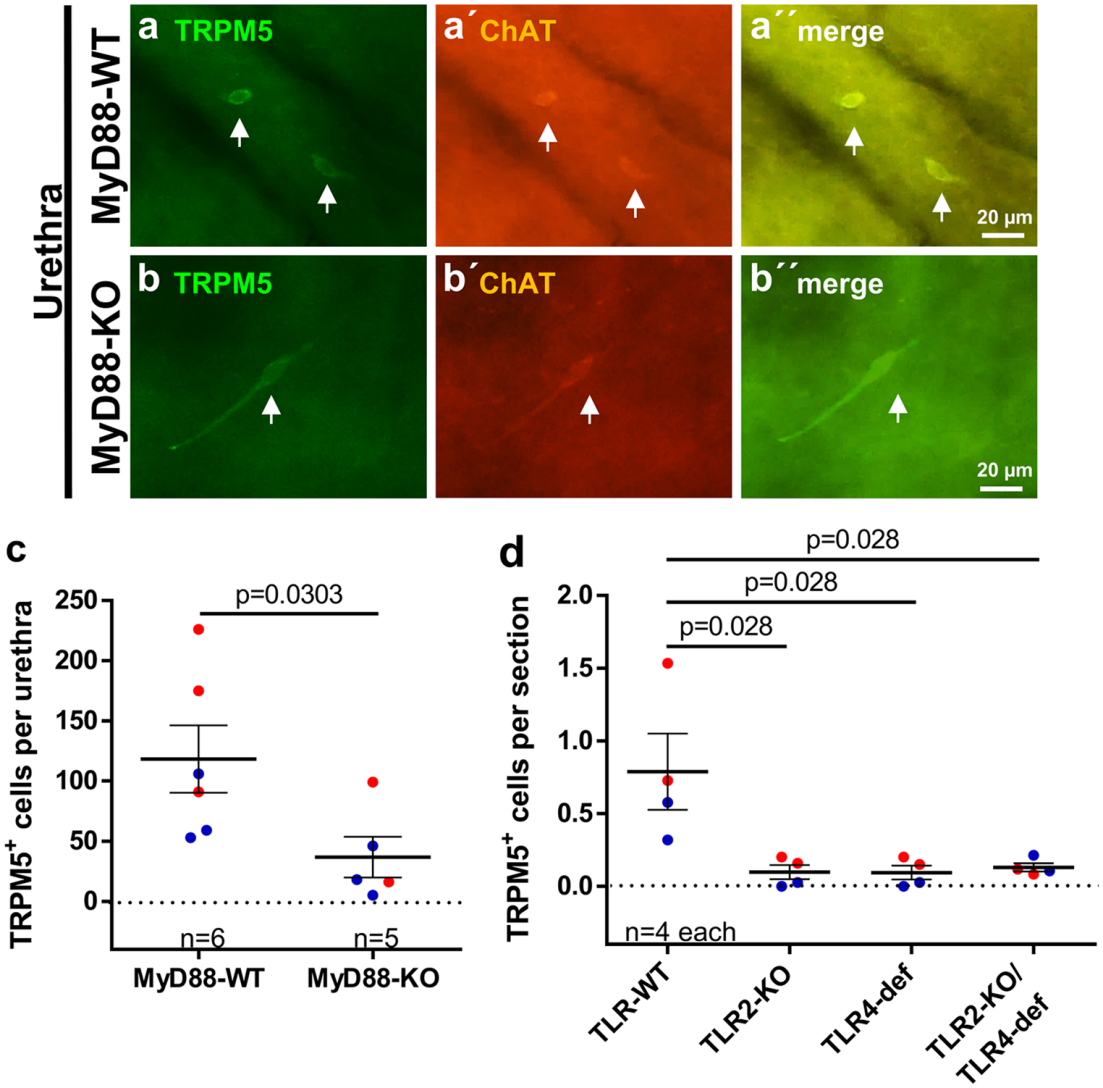
Urethral CCC in MyD88-KO, TLR2-KO, TLR4-def, and TLR2-KO/TLR4-def mice. **a** and **b** Representative pictures of TRPM5 and ChAT double-labeled CCC (arrows) in urethral whole-mounts of MyD88-WT **a** and MyD88-KO **b** mice; all examples taken from P100+ mice. **c** Quantitative analysis of TRPM5-positive cells per urethra counted at whole-mount preparations as shown in **a** and **b**. **d** Number of TRPM5-positive cells in urethral tissue sections of TLR2-KO, TLR4-def, TLR2-KO/TLR4-def, and TLR-WT mice. Each data point represents the average number of cells from 20 sections from one animal. Bars and whiskers depict mean and SEM. Blue: males, red: females. All investigated animals were adult (> 12 weeks). *P* values were calculated with Mann-Whitney test

We could not detect any urethral CCC in tissue sections from MyD88-KO mice stained with TRPM5 antibody (Supplementary Fig. 4a and b) and, hence, proceeded with whole-mount preparations using both TRPM5 and ChAT antibodies. TRPM5 antibody staining of urethral whole-mounts of adult ChAT-eGFP mice revealed about 99% congruency of ChAT-eGFP expression and TRPM5-immunolabeling antibody, justifying the use of TRPM5 antibody as a surrogate marker for urethral CCC (Supplementary Fig. 4c). Double staining of urethral whole-mount preparations from MyD88-KO mice and corresponding wildtypes with TRPM5 and ChAT antibodies also confirmed the near total coexistence of both signals (Fig. 4a and b). The total number of urethral CCC was significantly reduced by 69% in MyD88-KO mice compared with corresponding wildtypes (Fig. 4c). Since MyD88 is involved in downstream signaling of most TLRs (except TLR3), we reasoned that TLRs might be involved in the development of urethral CCC. We focused on TLR2 and TLR4 because of their importance in recognizing cell wall components of uropathogenic Gram-positive and -negative bacteria, respectively (Irvine et al. 2013; Park et al. 2009). Urethral sections from TLR2-KO, TLR4-def, and TLR2-KO/TLR4-def mice all showed significant reduction of TRMP5-immunoreactive cells by 88%, 88%, and 74%, respectively, when compared with corresponding wildtypes. However, no significant differences between the TLR-KO strains could be observed (Fig. 4d).

**Fig. 5:**
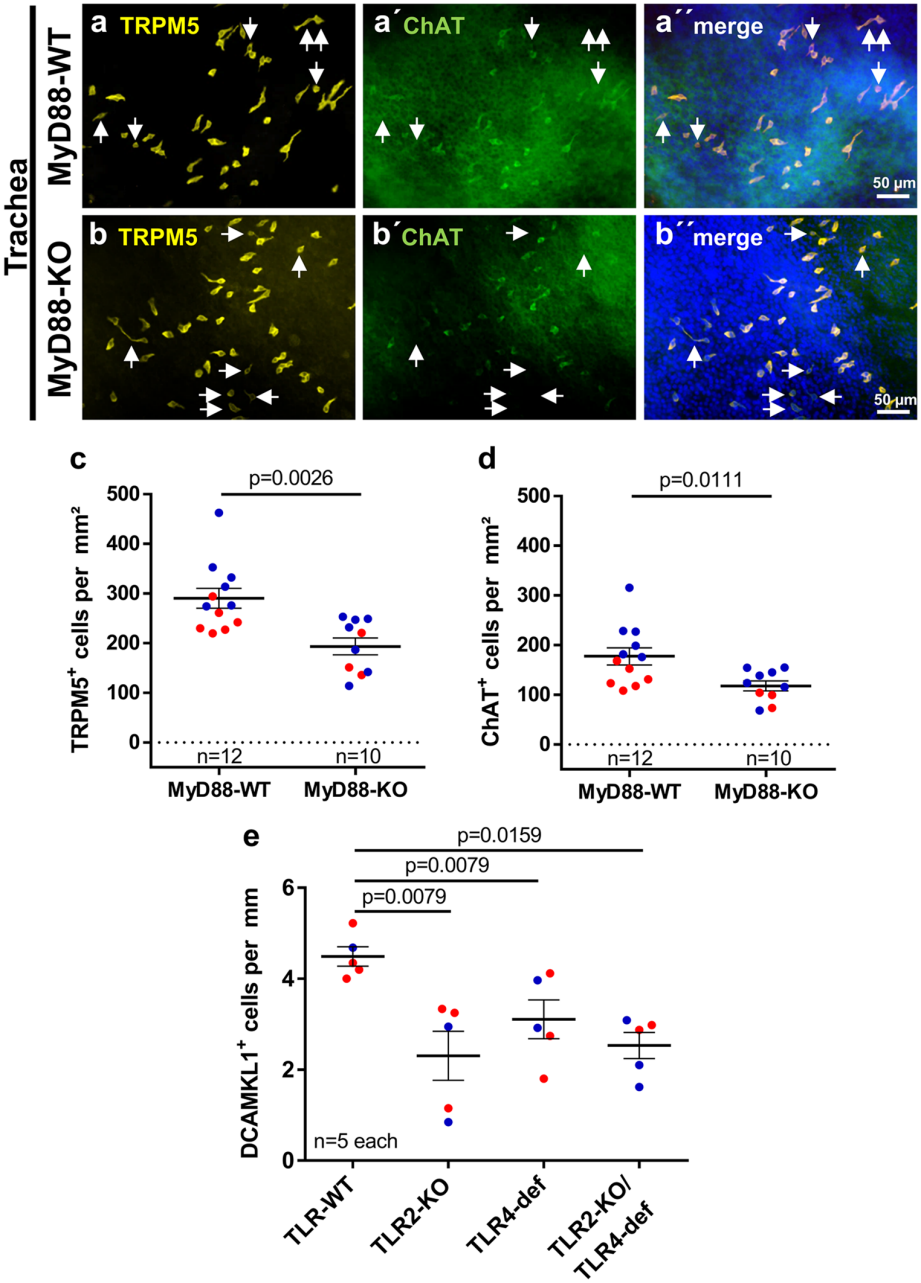
Tracheal CCC in MyD88-KO, TLR2-KO, TLR4-def, and TLR2-KO/TLR4-def mice. **a** and **b** Double-labeling immunofluorescence of tracheal whole-mounts of MyD88-WT **a** and MyD88-KO **b** mice with antibodies against TRPM5 (yellow) and ChAT (green). Double-labeled cells dominate, single-labeled CCC (TRPM5^+^/ChAT^−^) are marked with arrows. **c** and **d** Densities of TRPM5-positive cells **c** and ChAT-positive cells **c** counted from whole-mounts as shown in **a** and **b**. **e** Number of DCAMKL1-positive cells per mm of basal lamina in tracheal tissue sections of TLR2-KO, TLR4-def, TLR2-KO/TLR4-def, and TLR-WT mice. Each data point represents a single animal analyzed with an average number of 20 single sections. Bars and whiskers depict mean and SEM. Blue: males, red: females. All investigated animals were adult (> 12 weeks). *P* values were calculated with Mann-Whitney test

In tracheal whole-mount preparations, the congruency of staining with TRPM5 and ChAT antibodies was not 100%. TRPM5^+^/ChAT^−^ cells occurred in addition to double-labeled (TRPM5^+^/ChAT^+^) cells (Fig. 5a and b). Nevertheless, both stainings revealed significantly fewer CCC in MyD88-KO mice than in corresponding wildtypes (TRPM5: reduction by 36%, ChAT: reduction by 33%; Fig. 5c and d). Comparable results were also obtained with antibodies against DCAMKL1, another CCC marker, applied on TLR2-KO, TLR4-def, and TLR2-KO/TLR4-def tracheal sections (reduction of CCC by 47%, 29%, and 42%, respectively; Fig. 5e). DCAMKL1 was validated as a marker for CCC in tracheal whole-mount preparations of ChAT-eGFP mice, showing DCAMKL1-immunoreactivity in about 92% of all GFP-positive cells (Supplementary Fig. 5).

### CCC develop independent of living microbiota

**Fig. 6:**
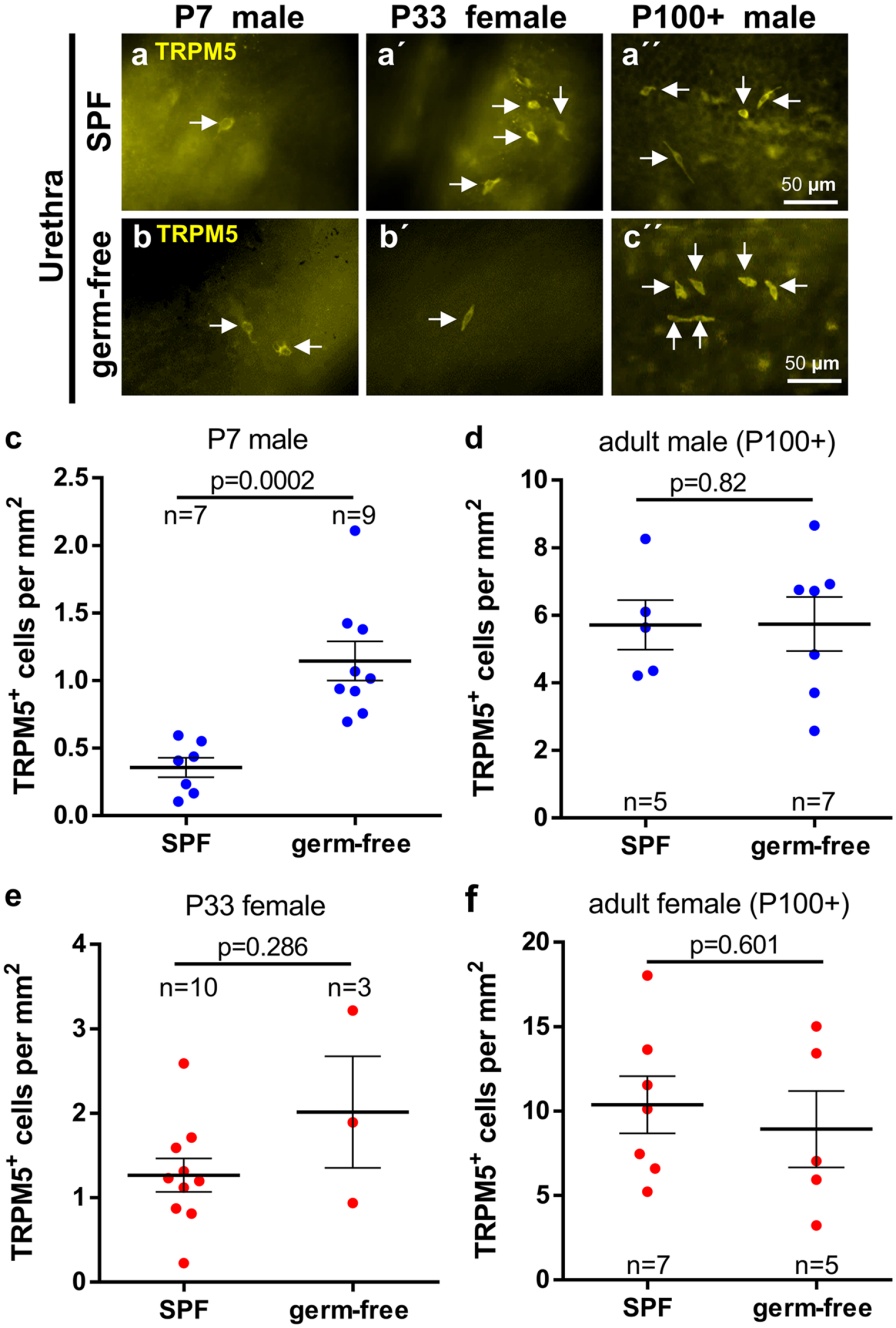
Postnatal development of urethral CCC in germ-free versus SPF mice. **a** and **b** Representative immunofluorescence pictures, TRPM5-immunolabeling of urethral whole-mounts of SPF **a** and germ-free **b** mice**.** **c**–**f** Quantitative analysis of whole-mounts shown in **a** and **b**. Bars and whiskers depict mean and SEM. Blue: males, red: females. All investigated animals were adult (> 12 weeks). *P* values were calculated with Mann-Whitney test

**Fig. 7:**
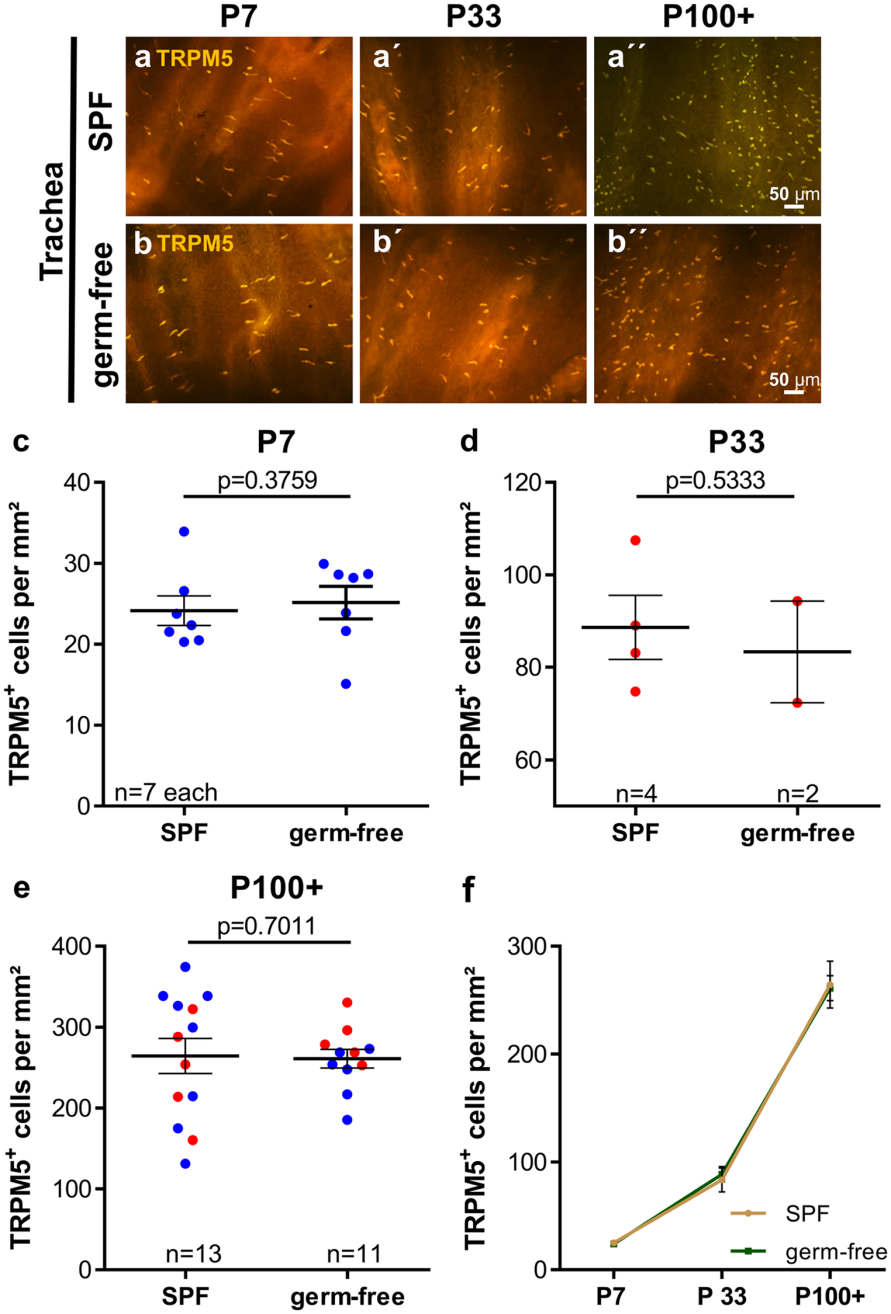
Postnatal development of tracheal CCC in germ-free versus SPF mice. **a** and **b** Representative immunofluorescence pictures, TRPM5-immunolabeling of tracheal whole-mounts. **c**–**e** Quantitative analysis of whole-mounts shown in **a** and **b**, **f** visualization of data shown in **c**–**e** over time. Graphs depict means and SEM. Blue: males, red: females. All investigated animals were adult (> 12 weeks). *P* values were calculated with Mann-Whitney test

To determine whether the presence of living microbiota has impact on the development of CCC, germ-free C57BL/6 N mice were investigated. Selection of investigated time points was guided by the preceding experiments on urethrae of ChAT-eGFP mice, showing initial appearance and average cell count of about 50 cells per urethra on P7 in male and P33 in female mice, and highest numbers in both sexes in mice older than P100 (Fig. 1b). TRPM5-immunolabeling was utilized to investigate postnatal development of urethral CCC in germ-free versus SPF mice (Fig. 6a and b). Significantly more TRPM5-positive urethral CCC were counted on P7 in germ-free male mice, compared with SPF-housed C57BL/6 N mice (Fig. 6c; Supplementary Fig. 6a), but neither in P33 female mice (Fig. 6e; Supplementary Fig. 6c) nor in adult (P100+) mice of either sex (Fig. 6d, f; Supplementary Fig. 6b, d). In the trachea, we could not detect any difference in cell number between germ-free and SPF-housed control mice, neither in young (P7 [male] and P33 [female]), nor in adult mice (both genders) (Fig. 7).

### Thymic CCC

In the course of our studies, we obtained some qualitative observations on thymic CCC. They were present already at birth (Supplementary Fig. 7), in adult MyD88-KO and TLR2/4-deficient mice (Supplementary Fig. 8), and in thymi of germ-free mice at the investigated time points, i.e., P7, P33, and P100+ (Supplementary Fig. 9).

## Discussion

The present data reveal a perinatal appearance and postnatal expansion of CCC with distinct organ-specific onset in development, sexual dimorphism, and dependency on pattern recognition receptor signaling. This time course of development renders a significant role of this cell type in intrauterine organ development unlikely, and rather points towards an onset of function in postnatal life. Accordingly, mucosal CCC are generally considered as sentinels monitoring luminal content for potentially harmful substances, predominantly of microbial origin (Deckmann and Kummer 2016; Finger and Kinnamon 2011; Krasteva and Kummer 2012; Kummer and Deckmann 2017; Schneider et al. 2019; Ting and von Moltke 2019; Tizzano and Finger 2013), to which exposure begins after birth. The present data also indicate at least a modulating and enhancing influence of the developing microbiota on the postnatal population dynamics of CCC, albeit microbiota are not an absolute requirement for the occurrence of CCC. This conclusion is based on our experiments with MyD88-KO and TLR-deficient mice. TLR2 and TLR4 are necessary for the detection of lipopeptides from bacterial origin and lipoteichoic acids or lipopolysaccharides from Gram-positive bacteria or Gram-negative bacteria, respectively, (Irvine et al. 2013; Park et al. 2009), and MyD88 is an essential downstream component of all TLR signaling except TLR3 (Wang et al. 2014). Genetic deletion of either of these components essentially reduced the number of CCC in the urethra and, to a lesser extent, in the trachea.

Likewise, MyD88 is also required for the induction of ChAT expression in lymphocytes (Reardon et al. 2013). Gnotobiotic mice are still exposed to TLR agonists, e.g., through their food and bedding material, and MyD88 expression was even found to be enhanced in the intestine of gnotobiotic mice compared with SPF mice (Yamamoto et al. 2012). This is consistent with our observation of generally unreduced and even enhanced numbers of CCC in male P7 urethra. These findings demonstrate that CCC development is independent of viability-associated pathogen-associated molecular patterns (vita PAMPs, (Sander et al. 2011)), but controlled by TLR2/4 signaling.

Although genetic deletion of TLR2, TLR4, TLR2/4, or MyD88 led to significantly reduced CCC numbers in the trachea, still more than 50% of CCC remained, pointing towards a CCC-inducing stimulus independent of pattern recognition signaling. This notion is supported by the presence of low basal numbers of CCC at birth (P0), prior to microbial contact. In line with our findings, early occurrence of chemosensory cells in the murine tracheal epithelium has also been described in TRPM5-GFP (P5, earlier time points not investigated; Saunders et al. 2013) and Tas2R131-GFP reporter mice (P3, earlier time points not investigated; Voigt et al. 2015). Similarly, we observed considerable numbers of CCC, albeit not quantified, in the thymic medulla of MyD88-KO, TLR2-KO/TLR4-def, and germ-free mice. They were also present at birth, consistent with the observation of Tas2R131-GFP^+^ and GNAT3-immunoreactive cells in the thymus at P3 (Voigt et al. 2015).

In contrast, genetic deletion of TLR2, TLR4, or MyD88 drastically reduced CCC numbers in the urethra by 69–88% compared with corresponding wildtypes and this coincided with lack of urethral CCC at birth. This strongly suggests that postnatal contact to TLR2/4 agonists, derived from the microbiome under physiological conditions, is required for CCC development in the urethra. MyD88 expression is regulated by estrogens (Zheng et al. 2006), and the downstream signaling proteins of the TLR4-MyD88 pathway, Bruton tyrosine kinase (BTK), and IL-1 receptor associated kinase (IRAK)1 are encoded by the X-chromosome (Spolarics 2007) with the consequence of higher IRAK1 levels in umbilical cord blood of female neonates compared with males (O'Driscoll et al. 2017). This has been considered one of the causes of sex-specific responses to infection and subsequent immunological advantage in female neonates (O'Driscoll et al. 2017). Notably, we also observed gender-specific differences in the first postnatal appearance of urethral CCC, which is also governed by TLR2/4-MyD88 signaling. It is tempting to assume a causal relationship, but this still needs to be experimentally addressed, in particular, since higher IRAK1 levels in females would predict earlier appearance of UCCC in females rather than in males. Consistent with this hypothesis, postnatal appearance and gender-specific developmental dynamics have also been reported for a closely related cell type, the brush cell of the rat common bile duct. It can be first detected by scanning electron microscopy at 4 weeks after birth and, thereafter, shows a remarkable increase in numbers, with a gender specific difference in time, i.e., between 8 and 12 weeks in the male and between 10 and 14 weeks in the female (Iseki 1991).

Collectively, our data show a marked postnatal expansion of CCC populations with distinct organ-specific features, including the relative impact of TLR2/4-MyD88 signaling. Strong dependency on this pathway (urethra) correlates with absence of CCC at birth and gender-specific initial development and expansion dynamics, whereas moderate dependency (trachea) coincides with presence of first CCC at E18 and sex-independent further development.

## Supplementary Information

Below is the link to the electronic supplementary material.

Supplementary file1 Supplementary Fig. 1 | Cell counting strategy on tracheal whole-mounts and tracheal surface area. (a) Representative tracheal whole-mount from a C57BL/6N mouse raised under SPF conditions (a control animal for comparison with germ-free mice) labelled with TRPM5-antibody (yellow); compound image obtained by stitching of 28 pictures; pictures from 8 areas (indicated by boxes) were taken, the cell number in each area was counted, and average cell number per picture and area calculated. (b) Tracheal surface area of ChAT-eGFP mice (N=2-8 each time point). (c) Numbers of ChAT-eGFP-positive cells per trachea in young animals (P0-8) and older animals (>P34) split by gender. Blue: males, red: females. Graph depicts mean and SEM. P-values were calculated with Mann-Whitney test. (TIF 10930 KB)

Supplementary file2 Supplementary Fig. 2 | Postnatal development of urethral CCC, aggregation of data shown in Fig. 1 in age groups. (a) ChAT-eGFP-positive cells per urethra in young animals (P6-39). (b and c) ChAT-eGFP-positive cells per mm2 in (b) young animals (P6-39) and (c) animals P40+; Bars and whiskers depict mean and SEM. Blue: males, red: females. P-values were calculated with Mann-Whitney test. (TIF 2927 KB)

Supplementary file3 Supplementary Fig. 3 | (a) Immunofluorescence pictures of tracheal whole-mounts used to generate quantitative data illustrated in Fig. 2b-e. Representative immunofluorescence pictures of tracheal whole-mounts of ChAT-eGFP mice at different ages (P0-108) with antibodies against TRPM5 (yellow) and eGFP (green). Solid arrows mark CCC which are double-positive for TRPM5 and ChAT-eGFP, dotted arrows mark cells which are only TRPM5-positive. (b) Immunofluorescence pictures of a tracheal whole-mount from an adult ChAT-eGFP mouse, strain originally described by von Engelhardt and colleagues (von Engelhardt et al. 2007) used in the prenatal and perinatal investigation of first appearance of CCC. (TIF 20365 KB)

Supplementary file4 Supplementary Fig. 4 | Urethral CCC in MyD88-KO, (a) Representative pictures of TRPM5-positive cells in urethral tissue sections of MyD88-KO and MyD88-WT mice, CCC are marked with arrows. (b) Quantitative analysis of TRPM5-positive cells in urethral tissue sections of MyD88-KO and MyD88-WT mice. Each data point represents a single section. CCC number was specified as cells per 1000 µm of basal membrane (BM). Total: MyD88-WT: 43 CCC in 17 sections from 8 animals with 35,237 µm basal membrane; MyD88-KO: 0 CCC in 11 sections from 5 animals with 28,282 µm basal membrane. Bars and whiskers depict mean and SEM. All tested animals were adult (>12 weeks). P-values were calculated with Mann-Whitney test. (c) Percentage of cells single positive for TRPM5 or ChAT-eGFP, or double positive for both in urethral whole mounts. (TIF 9412 KB)

Supplementary file5 Supplementary Fig. 5 | Validation of DCAMKL1 as a marker of tracheal CCC. (a) Representative immunofluorescence pictures of tracheal whole-mounts of a ChAT-eGFP mouse with antibodies against DCAMKL1 (yellow) and eGFP (green). Arrows mark cells which are single-positive for DCAMKL1, arrowheads mark cells which are ChAT-eGFP single-positive. (b) Relative frequencies of cells with DCAMKL1/ChAT-eGFP phenotypes +/+, +/- and -/+. Bars and whiskers depict means and SEM. Blue: males, red: females. All investigated animals were adult (>12 weeks). (TIF 6883 KB)

Supplementary file6 Supplementary Fig. 6 | Total numbers of TRPM5-immunoreactive cells in urethral whole-mounts of germ-free and control (SPF) male (a and b) and female (c and d) mice of different ages. Bars and whiskers depict mean and SEM. Blue: males, red: females. P-values were calculated with Mann-Whitney Test. (TIF 2977 KB)

Supplementary file7 Supplementary Fig. 7 | Thymic CCC at early postnatal stages. Double-labelling immunofluorescence (anti-TPRM5 and anti-GFP) of paraffin sections from thymi of ChAT-eGFP mice, double-labelled CCC (arrows) are already visible at birth (P0). Representative images of 3-5 samples investigated. (TIF 12029 KB)

Supplementary file8 Supplementary Fig. 8 | Thymic CCC in MyD88-KO (a) and TLR2-KO/TLR4-def mice (b). Representative immunofluorescence pictures with antibodies against TRPM5 (a) and DCAMKL1 (b). Arrows mark immunoreactive cells, autofluorescence of single cells, probably macrophages, is demonstrated using a 515-550 nm band pass barrier filter (asterisks in a). Representative images of 3-5 samples investigated per mouse strain. All investigated animals were adult (>12 weeks) (TIF 30006 KB)

Supplementary file9 Supplementary Fig. 9 | Thymic CCC in postnatal germ-free mice. Representative immunofluorescence pictures, TRPM5-immunolabelling (yellow, arrows indicate positive cells) and autofluorescence (green, asterisks indicate autofluorescent cells). Representative images of 3-5 samples investigated. (TIF 22250 KB)
