# Supplementary figures and images for: Development of epithelial cholinergic chemosensory cells of the urethra and trachea of mice
Source: Cell Tissue Res. 2021 Feb 22;385(1):21–35. doi: 10.1007/s00441-021-03424-9 (PMC8270884; doi:10.1007/s00441-021-03424-9)

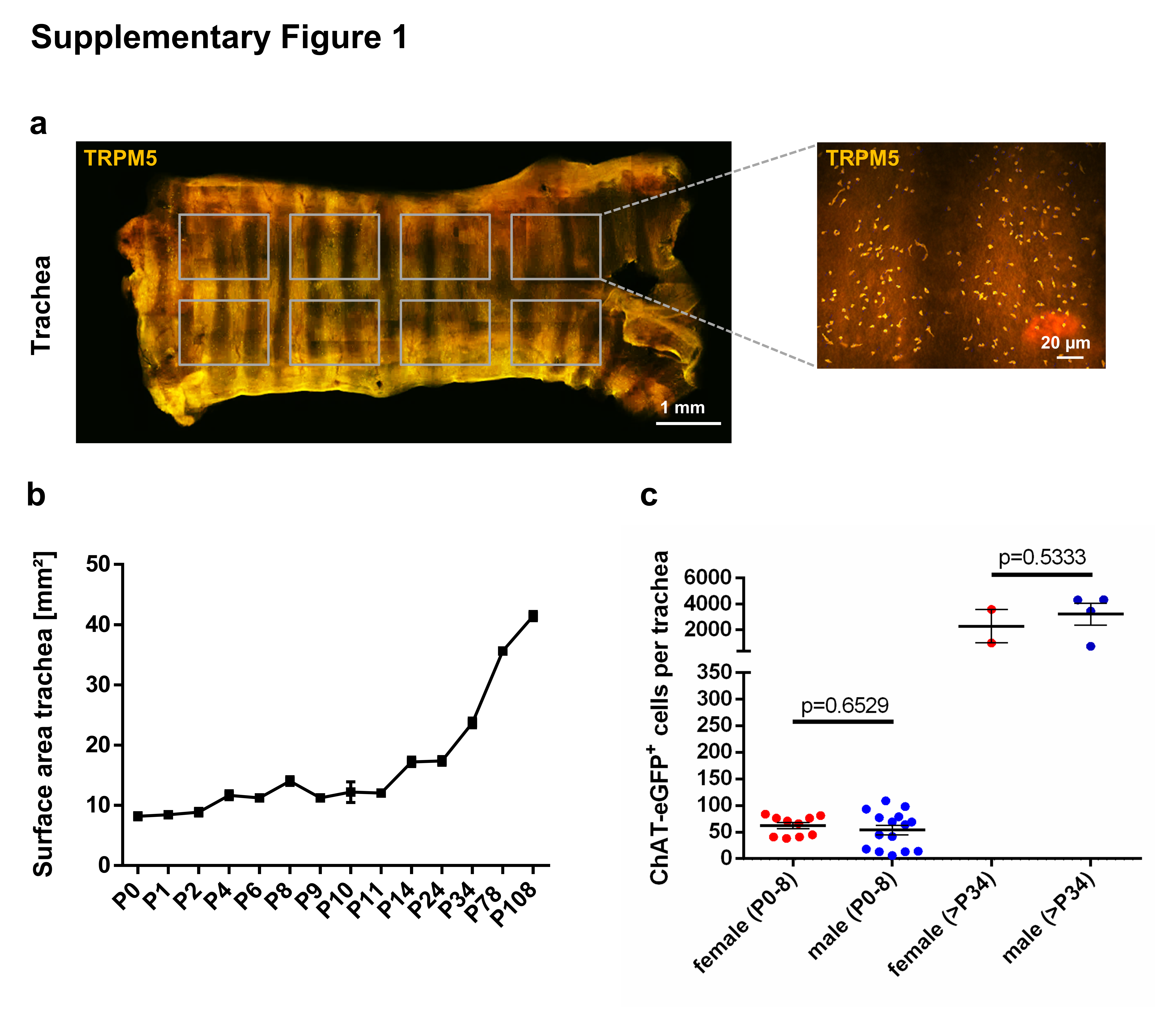

Supplement: Supplementary file 1 — Supplementary file1 Supplementary Fig. 1 | Cell counting strategy on tracheal whole-mounts and tracheal surface area. (a) Representative tracheal whole-mount from a C57BL/6N mouse raised under SPF conditions (a control animal for comparison with germ-free mice) labelled with TRPM5-antibody (yellow); compound image obtained by stitching of 28 pictures; pictures from 8 areas (indicated by boxes) were taken, the cell number in each area was counted, and average cell number per picture and area calculated. (b) Tracheal surface area of ChAT-eGFP mice (N=2-8 each time point). (c) Numbers of ChAT-eGFP-positive cells per trachea in young animals (P0-8) and older animals (>P34) split by gender. Blue: males, red: females. Graph depicts mean and SEM. P-values were calculated with Mann-Whitney test. (TIF 10930 KB) [file 441_2021_3424_MOESM1_ESM.tif]

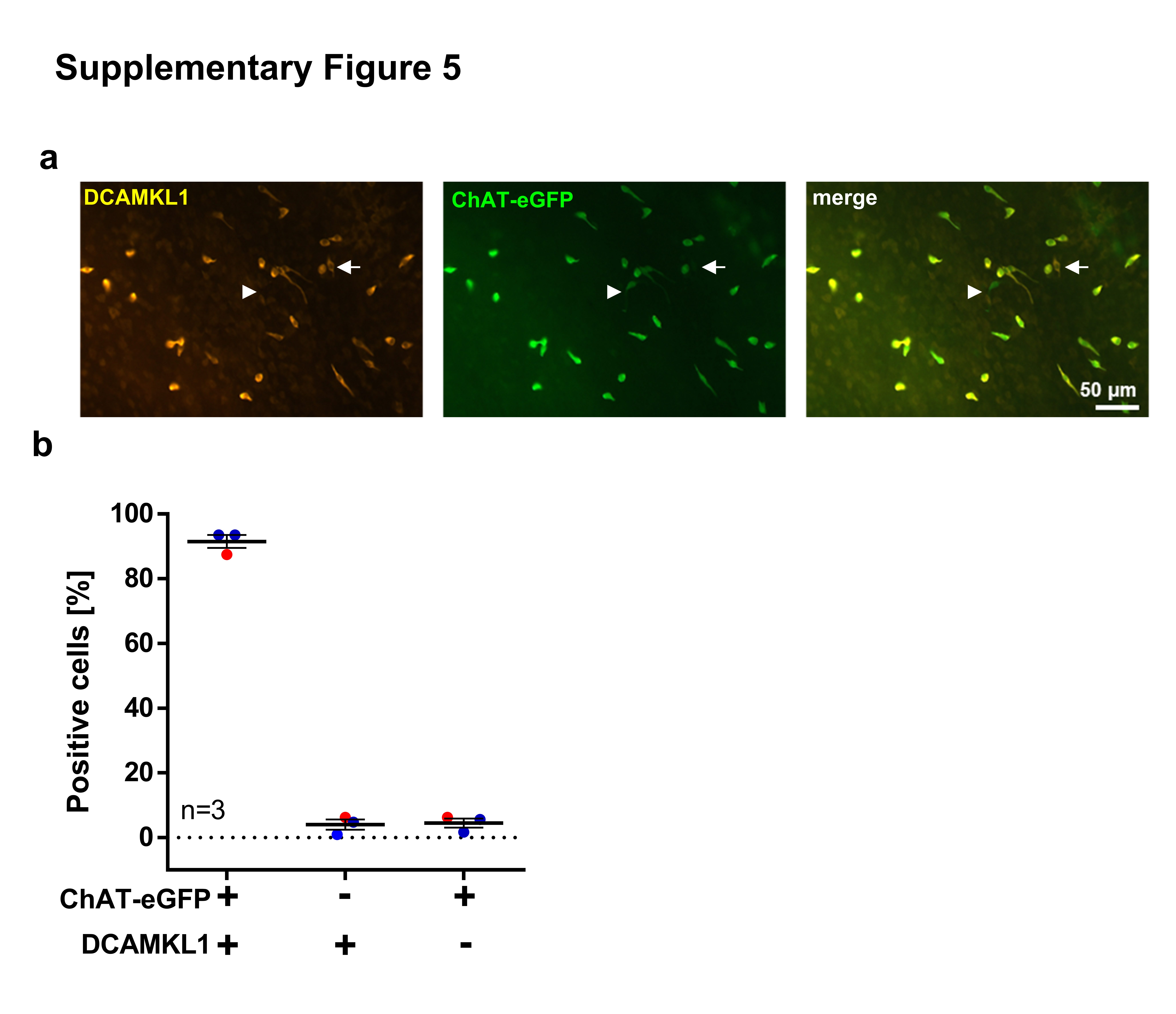

Supplement: Supplementary file 5 — Supplementary file5 Supplementary Fig. 5 | Validation of DCAMKL1 as a marker of tracheal CCC. (a) Representative immunofluorescence pictures of tracheal whole-mounts of a ChAT-eGFP mouse with antibodies against DCAMKL1 (yellow) and eGFP (green). Arrows mark cells which are single-positive for DCAMKL1, arrowheads mark cells which are ChAT-eGFP single-positive. (b) Relative frequencies of cells with DCAMKL1/ChAT-eGFP phenotypes +/+, +/- and -/+. Bars and whiskers depict means and SEM. Blue: males, red: females. All investigated animals were adult (>12 weeks). (TIF 6883 KB) [file 441_2021_3424_MOESM5_ESM.tif]

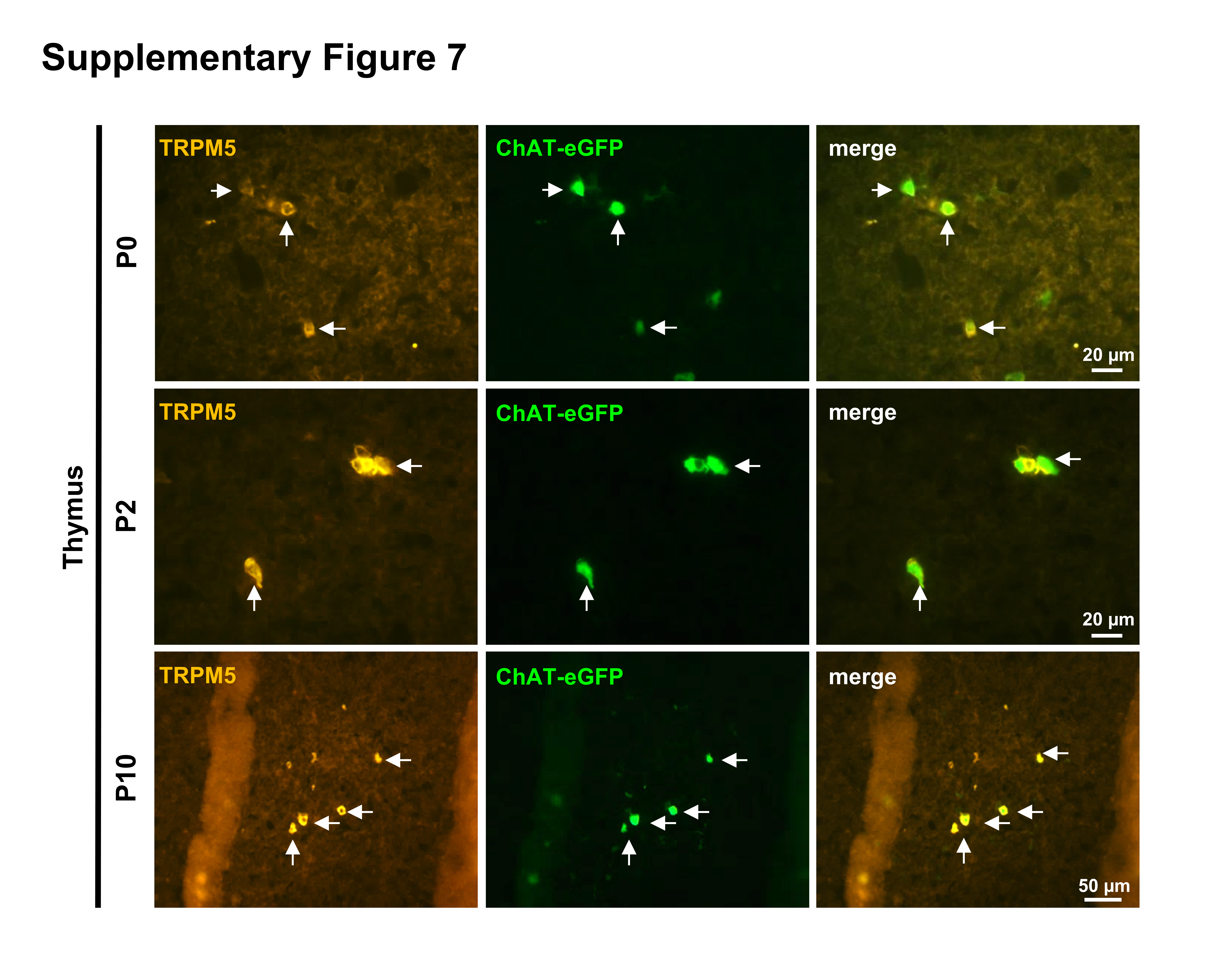

Supplement: Supplementary file 7 — Supplementary file7 Supplementary Fig. 7 | Thymic CCC at early postnatal stages. Double-labelling immunofluorescence (anti-TPRM5 and anti-GFP) of paraffin sections from thymi of ChAT-eGFP mice, double-labelled CCC (arrows) are already visible at birth (P0). Representative images of 3-5 samples investigated. (TIF 12029 KB) [file 441_2021_3424_MOESM7_ESM.tif]

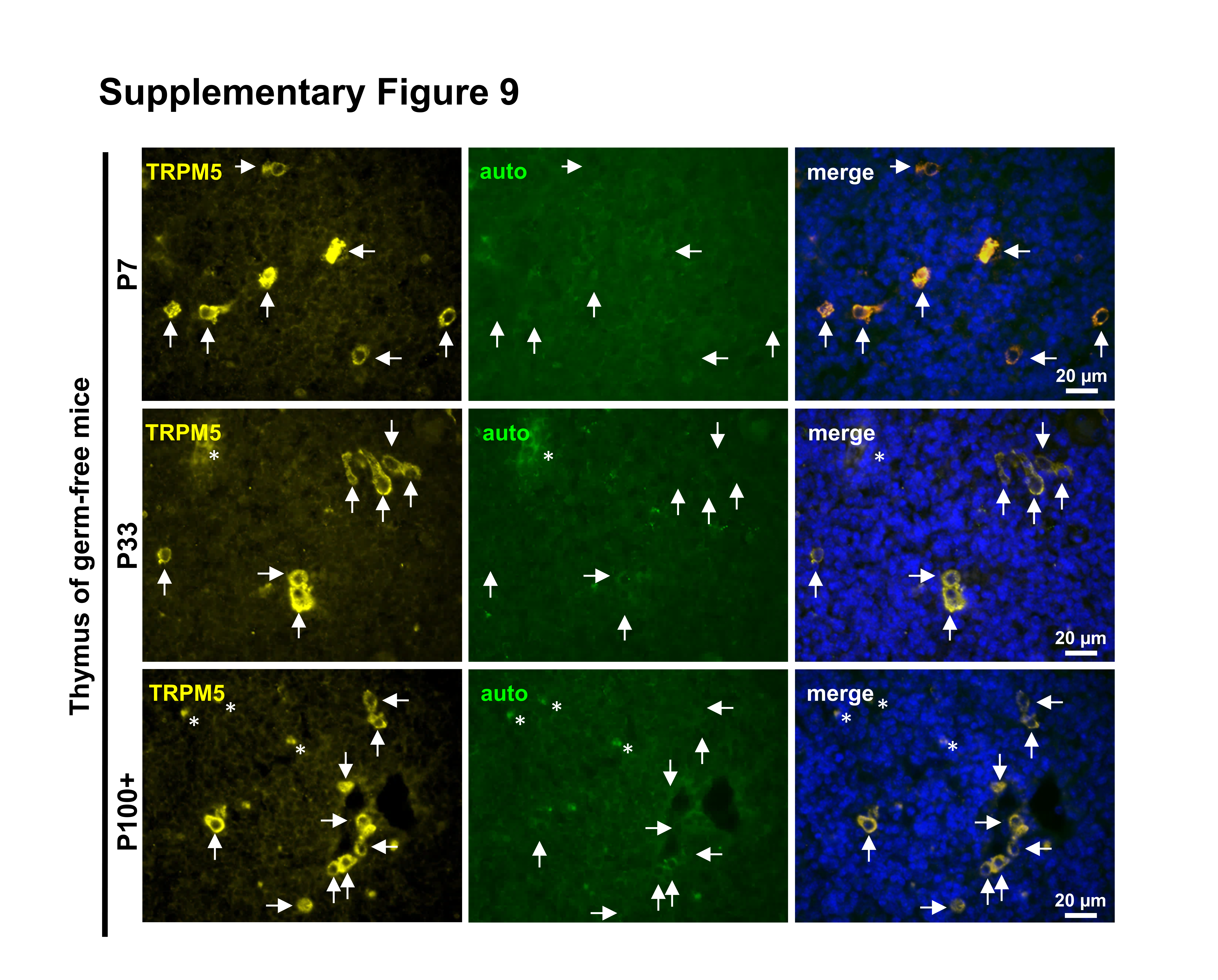

Supplement: Supplementary file 9 — Supplementary file9 Supplementary Fig. 9 | Thymic CCC in postnatal germ-free mice. Representative immunofluorescence pictures, TRPM5-immunolabelling (yellow, arrows indicate positive cells) and autofluorescence (green, asterisks indicate autofluorescent cells). Representative images of 3-5 samples investigated. (TIF 22250 KB) [file 441_2021_3424_MOESM9_ESM.tif]
